# Transcriptome-wide association study identifies novel genes associated with bone mineral density and lean body mass in children

**DOI:** 10.1007/s12020-022-03225-2

**Published:** 2022-12-27

**Authors:** Jiawen Xu, Jun Ma, Yi Zeng, Haibo Si, Yuangang Wu, Shaoyun Zhang, Bin Shen

**Affiliations:** Orthopedic Research Institute, Department of Orthopedics, Sichuan University West China Hospital, 37# Guoxue Road, Chengdu, 610041 Sichuan Province People’s Republic of China

**Keywords:** Transcriptome-wide association study (TWAS), Genome-wide association study (GWAS), Bone mineral density (BMD), Body lean mass (LM), Expression-trait association

## Abstract

**Objective:**

To identify novel candidate genes whose expression is associated with bone mineral density (BMD) and body lean mass (LM) in children.

**Methods:**

A tissue-specific transcriptome-wide association study (TWAS) was conducted utilizing a large-scale genome-wide association study (GWAS) dataset associated with BMD and LM and involving 10,414 participants. The measurement of BMD and LM phenotypes was made based on total-body dual-energy X-ray absorptiometry (TB-DXA) scans. TWAS was conducted by using FUSION software. Reference panels for muscle skeleton (MS), peripheral blood (NBL) and whole blood (YBL) were used for TWAS analysis. Functional enrichment and protein–protein interaction (PPI) analyses of the genes identified by TWAS were performed by using the online tool Metascape (http://metascape.org).

**Results:**

For BMD, we identified 174 genes with *P* < 0.05, such as IKZF1 (*P* = 1.46 × 10^−9^) and CHKB (*P* = 8.31 × 10^−7^). For LM, we identified 208 genes with *P* < 0.05, such as COPS5 (*P* = 3.03 × 10^−12^) and MRPS33 (*P* = 5.45 × 10^−10^). Gene ontology (GO) enrichment analysis of the BMD-associated genes revealed 200 GO terms, such as protein catabolic process (Log *P* = −5.09) and steroid hormone-mediated signaling pathway (Log *P* = −3.13). GO enrichment analysis of the LM-associated genes detected 287 GO terms, such as the apoptotic signaling pathway (Log *P* = −8.08) and lipid storage (Log *P* = −3.55).

**Conclusion:**

This study identified several candidate genes for BMD and LM in children, providing novel clues to the genetic mechanisms underlying the development of childhood BMD and LM.

## Introduction

Bone mineral density (BMD) is the amount of bone mineral in bone tissue and is often measured by using dual-energy X-ray absorptiometry (DXA) [1]. It is very widely used in clinical practice to assess the health status of bone in young people to identify those with osteoporosis and to evaluate the risk of fracture. Lean mass (LM) is the composition of our body and theoretically consists of muscle, organs, and bone [2]. LM can also be measured with DXA and is often thought to be a good proxy for skeletal muscle. Both bone and skeletal muscle are important components of the musculoskeletal system and are associated with the locomotion of our body.

The mechanisms of BMD and LM remain unclear, but it is thought to be multifactorial with complex interactions between genetic susceptibility and environmental factors. The heritability of BMD was estimated to be 50–80% [3]. Studies have shown that BMD has an obvious family genetic correlation. For example, Lutz et al. reported that the BMD of daughters is strongly correlated with premenopausal mothers [4]. In addition, several genome-wide association studies have been conducted to identify the susceptibility genes of BMD [5–13]. Over 60 different loci were identified to be robustly associated with BMD at different skeletal sites, including SREBF1, TOM1L2, WNT16, and LGR4 [14]. Meanwhile, the heritability of LM was estimated to be approximately 52% [14]. Family studies have demonstrated a significant familial resemblance for lean mass, suggesting that they are under genetic control [15]. However, the genetic mechanisms underlying BMD and LM are still elusive.

In classic Genome-wide association studies (GWASs), vast association signals have been detected between genetic variants and traits or phenotypes. In most cases, the association does not represent the real causal relationship partly because the mechanistic steps between genetic variants and traits or phenotypes cannot be taken into consideration. Furthermore, several lines of evidence show that a substantial proportion of risk variants exert their influence on traits by modulating the expression levels of the target genes (for example, in the case of expression quantitative trait loci (eQTLs)) [16, 17]. In this situation, it is needed to be solved the association between gene expression and traits. However, these types of studies have been hindered by difficulties in specimen collection and the high cost of assessing genotypes, phenotypes, and gene expression levels. To address these issues, transcriptome-wide association study (TWAS) was used to identify genes whose expression is associated with complex traits based on GWAS summary data [18].

Without directly measuring the gene expression levels, TWAS approach leverages a relatively small set of reference panels with both genotype and expression data to impute the gene expression levels in large-scale GWASs. Then, the imputed gene expression levels were used to identify the expression-trait association. Through this approach, several expression-trait associations have been found for various traits or diseases [18–22]. In recent years, TWAS approach has become a useful tool to evaluate the genetics of complex traits. For example, A. Gusev, et al. identified 157 TWAS-significant genes in a schizophrenia GWAS and found that one of the genes, mapk3, showed a significant effect on neurodevelopmental phenotypes in zebrafish [19]. There are several methods can be used in the TWAS, such as FUSION, PrediXcan, UTMOST, S-MultiXcan, et al. These methods can be divided in two groups: cross-tissue TWAS and single-tissue TWAS. Cross-tissue (MultiXcan, S-MultiXcan, and UTMOST) showed improved ability to identify gene-level associations in simulated and natural data compared to single-tissue TWAS (FUSION and PrediXcan). However, cross-tissue TWAS results were not tissue-specific, and therefore the cross-tissue TWAS could not reveal tissue-specific genetic regulatory mechanisms [23].

Here, we used FUSION software because it can estimate gene-trait associations and tissue-specificity. In other words, the genes identified by FUSION software are more tissue-specificity [18]. In this study, we report a tissue-specific TWAS based on a large GWAS dataset of BMD and LM. Reference panels for different tissues/cells, including muscle skeleton (MS), peripheral blood (NBL) and whole blood (YBL) panels, were used for imputation. Several significant expression-trait association signals were detected for both BMD and LM. Moreover, an enrichment analysis was performed to explore the functional annotations.

## Materials and methods

### GWAS summary data

The GWAS data used in our analysis were extracted from a previously published study [14] that enrolled 10,414 participants from four pediatric cohorts, namely, the Generation R Study, the Avon Longitudinal Study of Parents and their Children (ALSPAC), the Bone Mineral Density in Childhood Study (BMDCS), and the Copenhagen Prospective Studies on Asthma in Childhood (COPSAC) cohort. The average ages of the participants from the four cohorts varied in the range of 6.21–9.94 years. The measurements of BMD and LM phenotypes were made based on total-body dual-energy X-ray absorptiometry (TB-DXA) scans. DNA samples were genotyped using the Illumina platform. SNPs with a minor allele frequency (MAF) < 0.05 were excluded. Imputation to the CEU panel of the HapMap Phase II (build 36 release 22) reference panels was performed. BMD and LM measurements were adjusted by age, sex, height, fat percent (TB-FM/weight), and study-specific covariates (genetic principal components and measurement center). The GCTA software package and linkage disequilibrium-score regression methodology were used to assess the SNP heritability.

### TWAS analysis

TWAS was performed using FUSION software based on the pediatric GWAS summary data (http://gusevlab.org/projects/fusion/). The software provided an approach for identifying the association between complex traits and intermediate phenotypes (gene expression levels) without directly measuring the expression levels [18]. Given that the MS, NBL and YBL were used in previous biological studies of BMD and LM, the FUSION precomputed functional weights of gene expression of these three reference panels were used in our study [24–28]. Reference panels of different tissues were used to impute the cis genetic component expression of single nucleotide polymorphism (SNP) genotype data. In our study, the gene expression weights of the MS, NBL and YBL reference panel data, which were derived from FUSION, were used for the TWAS analysis. A *P* value was calculated for each analyzed gene. The significant association thresholds of MS should be *P* < 1.68 × 10^−5^ (0.05/2976), NBL should be *P* < 2.04 × 10^−5^ (0.05/2455) and YBL should be *P* < 1.06 × 10^−5^ (0.05/4701) after strict Bonferroni correction. *P* values between 1.68 × 10^−5^ (for MS, 2.04 × 10^−5^ for NBL, and 1.06 × 10^−5^ for YBL) and 0.05 were considered to be suggestive of significance.

### Functional annotation and enrichment analysis

To better interpret the associated genes identified in the TWAS, the online tool Metascape was used to perform further functional enrichment analysis and protein–protein interaction (PPI) analysis (http://metascape.org) [29]. A standard accumulative hypergeometric statistical test was applied to identify ontology terms based on canonical pathway (MSigDB), hallmark gene set (MSigDB), Kyoto Encyclopedia of Genes and Genomes (KEGG) pathway and gene ontology (GO) biological processes resources. The construction of the PPI network and associated module analysis were based on GO enrichment analysis using the plugin Molecular Complex Detection (MCODE). The MCODE algorithm was then applied to this network to identify neighborhoods where proteins are densely connected. Based on Bonferroni correction, the significance thresholds were Log *P* < −3.60 for BMD and Log *P* < −3.75 for LM. Log *P* values between Log *P* −3.60/Log *P* −3.75 and Log *P* −1.30 were considered to be suggestive of significance.

## Results

### Associated genes identified by the TWAS

**Fig. 1:**
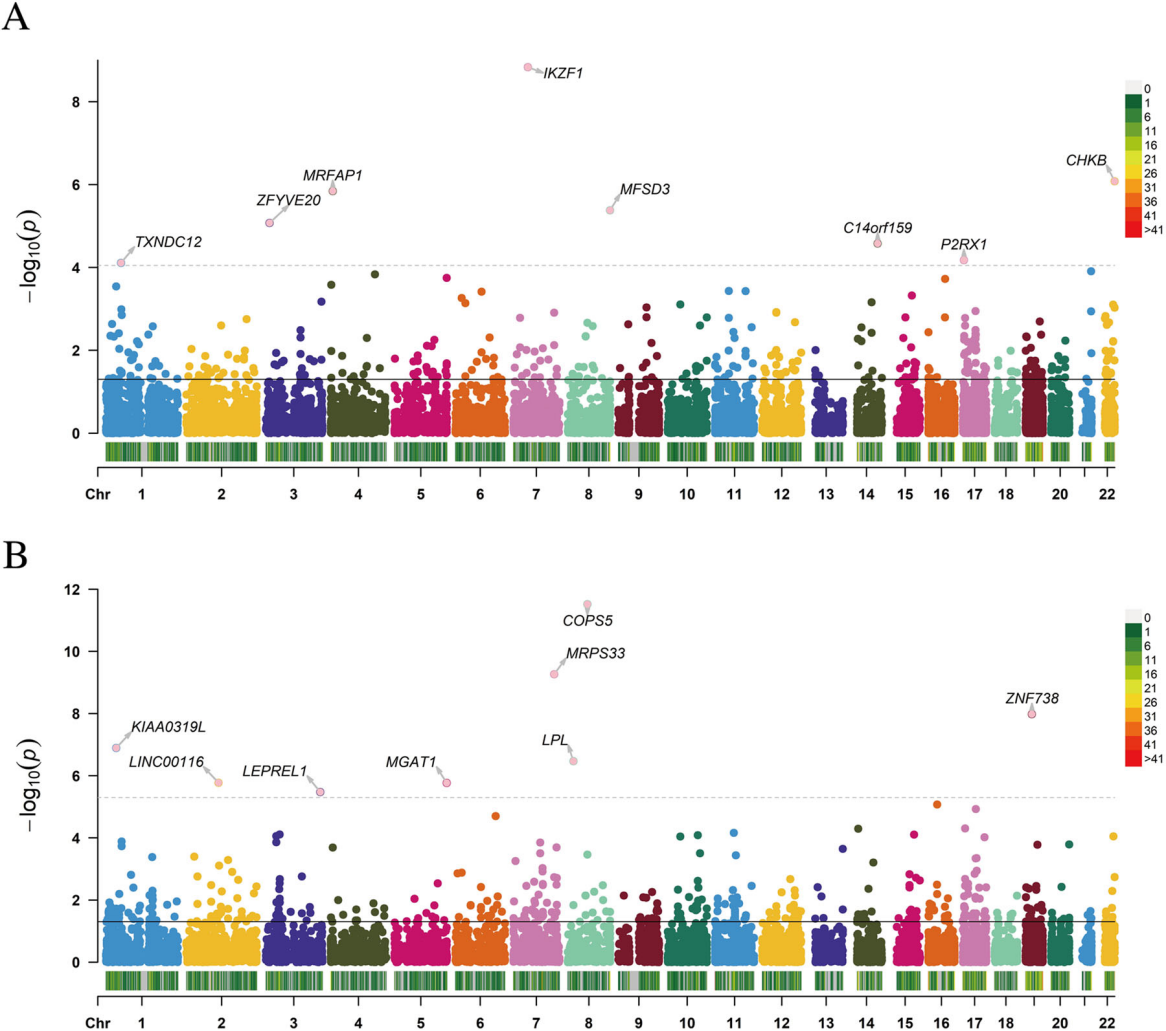
Manhattan plot showing TWAS-identified genes. Manhattan plot showing TWAS-identified genes and significantly expressed genes associated. with bone mineral density (**A** BMD) and lean mass (**B** LM). Each point represents a single gene, and the physical position (chromosome localization) is plotted on the *x*-axis, while the -log10 (*P* value) of the association between gene and BMD or LM is plotted on the *y*-axis. TWAS Transcriptome-wide association study, BMD bone mineral density, LM lean mass

For different tissues/cells of the MS, NBL, and YBL panels, 2976, 2455, and 4701 genes were finally included in the expression-training association analysis (Table 1). For the childhood BMD phenotype, TWAS identified 120, 86, and 174 genes with *P* < 0.05 based on the MS, NBL, and YBL panels, such as IKZF1 (*P* = 1.46 × 10^−9^) and CHKB (*P* = 8.31 × 10^−7^). For childhood LM, we detected 145, 94, and 208 genes with *P* < 0.05 based on the MS, NBL, and YBL panels, such as COPS5 (*P* = 3.03 × 10^−12^) and MRPS33 (*P* = 5.45 × 10^−10^) (Fig. 1). The 10 top significant genes in the BMD and LM groups are shown in Table 2, including population, heritability of genes (HSQ), and the BEST.GWAS. ID, number of SNPs in the locus (NSNP), and TWAS P value (P TWAS).

**Table 1:** The number of associated with BMD and LM identified by the TWAS

Tissue/cell	*N*^#^	*n*^*#*^ for BMD	*n*^*#*^ for LM
MS	2976	120	145
NBL	2455	86	94
YBL	4701	174	208

*BMD* bone mineral density, *LM* lean mass, *TWAS* transcriptome-wide association study

*N* means the number of genes finally included in the analysis of TWAS

*n* means the number of significantly associated genes identified in the TWAS

**Table 2:** Top genes selected by TWAS analysis (BMD and LM)

	Gene	CHR	BEST.GWAS.ID	NSNP	TWAS.Z	TWAS.P
BMD	IKZF1	7	rs7779747	534	−6.0490	1.46E-09
	CHKB	22	rs761744	263	−3.9353	8.31E-07
	MRFAP1	4	rs7660424	493	4.8190	1.44E-06
	MFSD3	8	rs6558318	186	4.6027	4.17E-06
	ZFYVE20	3	rs17040623	490	4.4536	8.44E-06
	C14orf159	14	rs1286341	450	4.2027	2.64E-05
	P2RX1	17	rs17822998	463	3.9892	6.63E-05
	TXNDC12	1	rs6686632	194	3.9516	7.76E-05
	C21orf89	21	rs2838846	522	−3.8387	1.24E-04
	RPS3A	4	rs7657668	264	3.7963	1.47E-04
LM	COPS5	8	rs16933079	177	6.9761	3.03E-12
	MRPS33	7	rs10488014	378	−6.2056	5.45E-10
	ZNF738	19	rs661453	288	−5.7224	1.05E-08
	KIAA0319L	1	rs6668101	215	5.2800	1.28E-07
	LPL	8	rs1569209	679	5.1003	3.39E-07
	LINC00116	2	rs11904760	72	4.7872	1.69E-06
	MGAT1	5	rs3733754	350	−4.7852	1.71E-06
	LEPREL1	3	rs6788300	652	−4.6479	3.35E-06
	CD2BP2	16	rs7196298	191	−4.4548	8.40E-06
	WNT3	17	rs16941702	253	−4.3818	1.18E-05

The large-scale Genome-Wide Association Study (GWAS) summary data for BMD and LM acquired from a cohort study, including 10,414 participants. The TWAS.P and TWAS.*Z* values were calculated by the FUSION approach (http://gusevlab.org/projects/fusion/)

*TWAS* Transcriptome-Wide Association Study, *GWAS* Genome-Wide Association Study, *BMD* Bone mineral density, *LM* Lean mass, *TWAS P* TWAS *P* value, *TWAS Z* TWAS *Z*-score, *HSQ* heritability of genes, *NSNP* number of SNPs in the locus

### Functional enrichment and PPI analysis results

**Fig. 2:**
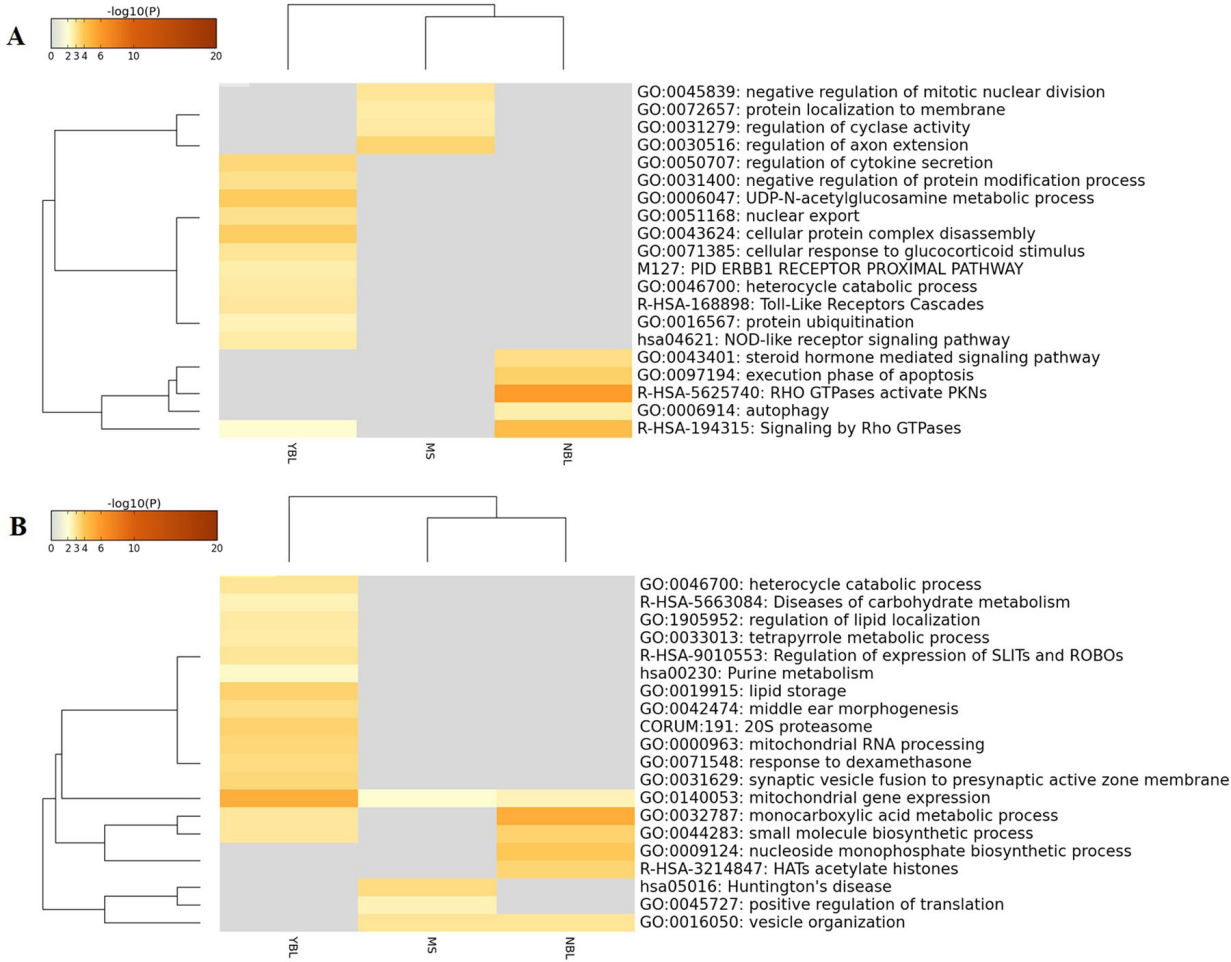
The heatmap of enriched terms of BMD (**A**) and LM (**B**). The heatmap showed the enriched terms of bone mineral density (**A** BMD) and lean mass (**B** LM). BMD bone mineral density, LM lean mass

**Fig. 3:**
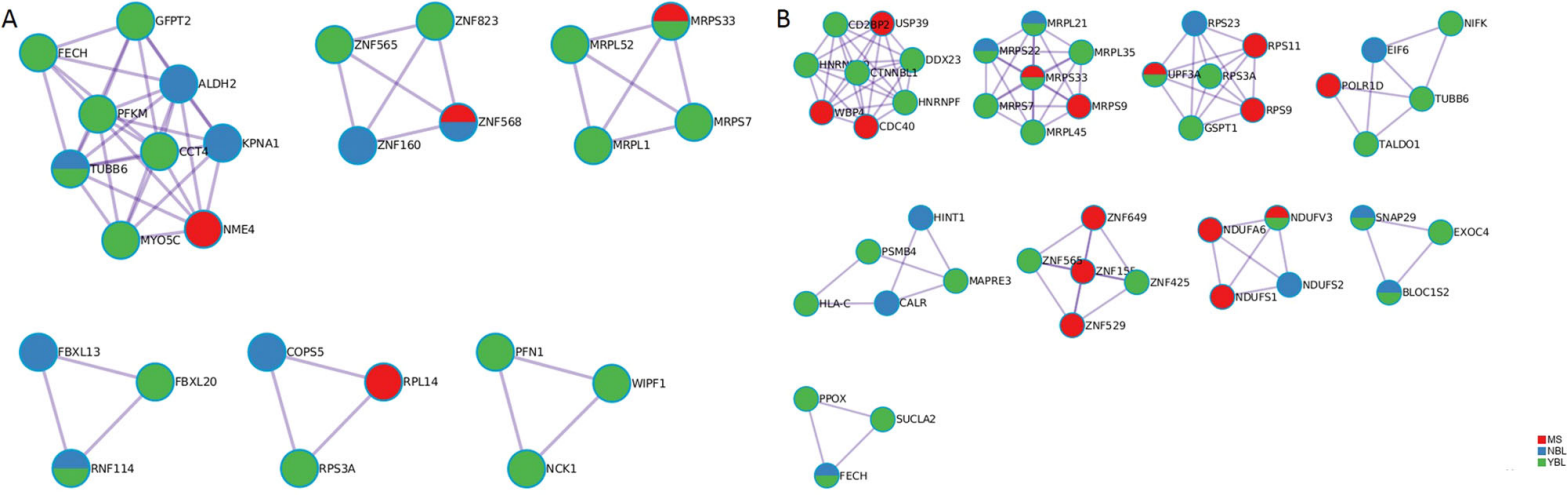
Protein-protein interaction (PPI) enrichment analysis of BMD (**A**) and LM (**B**). Protein-protein interaction (PPI) enrichment analysis was also carried out with the following databases: BioGrid7, InWeb_IM8, OmniPath9. PPI Protein-protein interaction, BMD bone mineral density, LM lean mass

In this study, functional enrichment analysis was carried out with the following ontology sources: GO biological processes, KEGG pathways, GO molecular functions, reactome gene sets, canonical pathways, and CORUM. The heatmap of the enriched terms is shown in Fig. 2. The enrichment analysis results showed that the genes significantly associated with BMD were enriched in hormone-related categories, such as the apoptotic signaling pathway (Log *P* = −8.08), steroid hormone-mediated signaling pathway (Log *P* = −3.13), regulation of growth hormone secretion (Log *P* = −3.43) and cellular response to glucocorticoid stimulus (Log *P* = −2.95). For LM, it seems that the associated genes are more likely to be enriched in the metabolism categories of several substances, including protein catabolic process (Log *P* = −5.09), carbohydrate metabolism (Log *P* = −2.43), regulation of lipid localization (Log *P* = −2.80), and lipid storage (Log *P* = −3.55). PPI enrichment analysis was also carried out with the following databases: BioGrid7, InWeb_IM8, and OmniPath9. The MCODE networks identified for individual gene lists were pooled and are shown in Fig. 3.

## Discussion

BMD and LM are complex traits that can be influenced by multiple genetic factors. Although previous studies have reported various genes associated with the two traits, limited mechanistic information was provided in these studies. Genetic variants can influence traits by regulating gene expression levels. Therefore, we conducted this tissue-specific TWAS to investigate the expression-trait associations for BMD and LM. Databases with both expression levels and genotype data for tissues/cells, namely, MS, NBL, and YBL, were used as reference panels for expression level imputation.

SREBF1 (sterol regulatory element-binding factor 1), a significant gene we identified associated with both BMD and LM, is consistent with the results reported in previous studies [14]. The active product of SREBF1, SREBP-1, regulates muscle protein synthesis by downregulating the expression of MYOD1, MYOG, and MEF2C factors, while overexpression inhibits protein synthesis and thus induces myotubular atrophy [30, 31]. In addition, decreased expression of SREBP-1 also leads to decreased NF-κB signaling, which in turn decreases osteoclast formation and bone resorption activity, while enhanced expression of SREBF, in contrast, enhances osteoblast mineralization and thus increases BMD [32]. Overall, the biological activity of SREBF is pleiotropic, with opposite effects on LM and BMD occurrence.

By using TWAS analysis, several genes, such as COPS5, were identified with LM. COP9 signaling vesicle complex 5 (COPS5) is an important member of the ubiquitin‒proteasome system that plays an important role in ubiquitin-mediated protein degradation and is also involved in the regulation of cell development [33, 34] COPS5 regulates the myosin-interacting proteins UNC-98 and UNC96 to modulate muscle protein levels [35]. Velardo et al. showed that COPS5 plays an important role in muscle development, maintenance and regeneration and is associated with the development of congenital muscular dystrophy [36]. In addition, COPS5 is necessary for osteoblast development and postnatal bone formation, and it may function by modulating the TGF and BMP signaling pathways in osteoblast progenitor cells [37]. Thus, COPS5 may affect LM by regulating muscle and bone metabolism.

For BMD, TWAS analysis also identified several genes, such as IKZF1 and CHKB. Choline kinase β (CHKB), a kinase involved in phosphatidylcholine biosynthesis, is a gene that we have identified as significantly associated with BMD. A study demonstrated that CHKB deficiency results in defective formation and function of osteoclasts and osteoblasts, leading to reduced bone mass, and is considered to be a regulator of bone homeostasis [38]. CHKB activation may influence osteoclastic bone resorption by influencing the gene expression of chloride and voltage-dependent calcium channels that are required for osteoclast activity [39, 40]. Recent studies have shown that CHKB deficiency leads to congenital myotonic dystrophy, including microcephaly and facial dysmorphism [41]. Therefore, CHKB activity and BMD are closely related, but the exact mechanism of action needs to be further investigated.

The enrichment analysis detected 200 terms associated with BMD, such as apoptotic signaling pathway (GO: 0097190, Log *P* = −8.08), steroid hormone mediated signaling pathway (GO: 0043401, Log *P* = −3.13), autophagy (GO: 0006914, Log *P* = −2.65), regulation of cytokine secretion (GO: 0050707, Log *P* = −3.43) and cellular response to glucocorticoid stimulus (GO: 0071385, Log *P* = −2.95). By using enrichment analysis, we found that the apoptotic signaling pathway had the most relevant association with BMD. Apoptosis plays an important role in bone growth, bone remodeling, and bone regeneration, and increased osteocyte apoptosis is often associated with rapid bone turnover [42]. According to studies, excessive use of glucocorticoids or aging can accelerate osteoblast apoptosis and result in bone loss [43, 44]. In addition, studies have demonstrated that postmenopausal women have decreased estrogen secretion, which stimulates osteoblasts to secrete cytokines such as NF-κB that activate downstream apoptotic pathways and inhibit osteoblast proliferation and differentiation, resulting in decreased bone mineral density and osteoporosis [45]. In addition, these pathways were most enriched in the hormone-related categories. Steroid hormones such as glucocorticoids were associated with decreased BMD and impaired bone microarchitecture parameters [46]. These findings seem consistent with previous knowledge of BMD.

For LM, enrichment analysis detected 287 terms that were most likely to be enriched in the metabolism categories of several substances, including protein catabolic process (GO:0030163, Log *P* = −5.09), diseases of carbohydrate metabolism (R-HSA-5663084, Log *P* = −2.43), regulation of lipid localization (GO: 1905952, Log *P* = −2.80), lipid storage (GO: 0019915, Log *P* = −3.55) and mitochondrial RNA processing (GO: 0000963, Log *P* = −3.41). Muscle atrophy is the most significant symptom of LM, and we found that the protein catabolic process is the most relevant pathway associated with LM. The ubiquitin‒proteasome system is the best-known cellular protein degradation system responsible for the hydrolysis of damaged proteins collected in skeletal muscle, and multiple studies have demonstrated that UPS overexpression results in skeletal muscle atrophy [47]. In addition, during famine, aging, and excessive glucocorticoid use, the glucocorticoid receptor and transcription factor forkhead box O increase the expression of MuRF1 and accelerate protein catabolism, ultimately resulting in muscle atrophy [48]. In contrast, protein supplementation can successfully prevent muscle atrophy by reversing the imbalance between protein catabolism and anabolism [49]. In addition, adequate research has demonstrated that reduced carbohydrate and lipid consumption leads to lower body weight by increasing lipid oxidation [50, 51]. Compared with BMD, LM is more likely to be regulated by biochemical metabolism.

According to the PPI network, aberrant expression of ribosomal protein (RP) is intimately linked to BMD and LM. RP refers to the proteins that comprise the ribosome and serve a key regulatory function in ribosome biosynthesis, peptide bond formation, and protein synthesis [52]. During the growth and development of bones, ribosome-mediated protein synthesis is necessary for the formation of bone matrix. According to studies, aberrant expression of some RP proteins may be linked to congenital skeletal growth abnormalities and bone marrow failure [53]. Studies on animals indicate that mice with impaired ribosome function have changed rates of matrix protein synthesis, resulting in delayed bone formation, decreased bone mass, and heightened bone fragility [54]. In addition to RPs, ZNF823, ZNF565, ZNF160, and ZNF568 are also aberrant regulatory proteins screened by the PPI network. Schnurri-3, a zinc finger protein, was demonstrated to modulate osteoclast activity to modify bone mass [55].

The TWAS analysis approach developed by Alexander G et al. was adopted in this study [18]. It can be used to identify genes whose expression is significantly associated with complex traits in individuals based on GWAS summary data without directly measuring the expression levels of these genes [18]. There are several potential advantages of using this approach. First, the expression-trait association information can provide more interpretable insights for further genetic studies, while GWASs often obtain associated loci lying in the linkage disequilibrium with multiple significant SNPs that may not be in the genes. Moreover, unlike analyses focusing on eQTL and SNP associations, TWASs can combine full cis-SNP signals, regardless of whether they are significant, to make an expression level imputation. Finally, confounding from environmental differences caused by the traits can be avoided in TWAS analyses.

There are also some limitations of this study that must be addressed. First, as the gene expression level is imputed based on the reference panels, there is a possibility that the results can be influenced by the quality and sample size of the reference data. A larger sample size and more available tissue datasets can mitigate this issue. Second, the number of imputed genes depends on the training data. Therefore, it is limited to some degree. Finally, after multiple testing and correction, the significance threshold of enrichment analysis was Log *P* < −3.60 for BMD and Log *P* < −3.75 for LM. Unfortunately, according to our results, some terms enriched in this study, such as steroid hormone-mediated signaling pathways and diseases of carbohydrate metabolism, showed suggestive associations with BMD and LM. Therefore, the results of the suggested terms should be interpreted carefully.

## Conclusion

This study was a tissue-specific TWAS of the phenotypes of BMD and LM based on previous GWAS datasets. The expression levels of several genes were identified to be associated with BMD and LM. By performing gene enrichment analysis, we found that the two traits had different regulatory mechanisms. This study could provide novel insights for the further study of BMD and LM and an outline for a systematic approach for identifying functional mediators of complex disease.

## Data Availability

The GWAS summary dataset was extracted from a previously published study (Bivariate genome-wide association analysis implicates pleiotropic effects at the SREBF1/TOM1L2 locus on bone mineral density and lean mass in children).
